# Phenotypic and Functional Comparison of Class Switch Recombination Deficiencies with a Subgroup of Common Variable Immunodeficiencies

**DOI:** 10.1007/s10875-016-0321-2

**Published:** 2016-08-02

**Authors:** Daan J. aan de Kerk, Machiel H. Jansen, Stephen Jolles, Klaus Warnatz, Suranjith L. Seneviratne, Ineke J. M. ten Berge, Ester M. M. van Leeuwen, Taco W. Kuijpers

**Affiliations:** 1 Department of Pediatric Hematology, Immunology and Infectious Diseases, Academic Medical Center (AMC), Amsterdam, The Netherlands; 2 Department of Experimental Immunology, AMC, Amsterdam, The Netherlands; 3 Department of Immunology, University Hospital of Wales Heath Park Cardiff, Cardiff, UK; 4 Department of Rheumatology and Clinical Immunology, University Medical Centre Freiburg, Freiburg, Germany; 5 Department of Immunology, Royal Free London NHS Foundation Trust, London, UK; 6 Department of Internal Medicine, AMC, Amsterdam, The Netherlands

**Keywords:** Primary antibody deficiencies, class switch recombination defect, common variable immunodeficiency, B cells

## Abstract

Primary antibody deficiencies (PADs) are the most common immunodeficiency in humans, characterized by low levels of immunoglobulins and inadequate antibody responses upon immunization. These PADs may result from an early block in B cell development with a complete absence of peripheral B cells and lack of immunoglobulins. In the presence of circulating B cells, some PADs are genetically caused by a class switch recombination (CSR) defect, but in the most common PAD, common variable immunodeficiency (CVID), very few gene defects have as yet been characterized despite various phenotypic classifications. Using a functional read-out, we previously identified a functional subgroup of CVID patients with plasmablasts (PBs) producing IgM only. We have now further characterized such CVID patients by a direct functional comparison with patients having genetically well-characterized CSR defects in CD40L, activation-induced cytidine deaminase (AID) and uracil N-glycosylase activity (UNG). The CSR-like CVID patients showed a failure in B cell activation patterns similar to the classical AID/UNG defects in three out of five CVID patients and distinct more individual defects in the two other CVID cases when tested for cellular activation and PB differentiation. Thus, functional categorization of B cell activation and differentiation pathways extends the expected variation in CVID to CSR-like defects of as yet unknown genetic etiology.

**Electronic supplementary material:**

The online version of this article (doi:10.1007/s10875-016-0321-2) contains supplementary material, which is available to authorized users.

## Introduction

Primary immunodeficiencies (PIDs) are well recognized in the Western world [1–3]. About 65 % of these PIDs show an altered antibody production, categorized as primary antibody deficiency (PAD). PADs can be caused by either a combined T and B cell defect or a B cell-intrinsic defect. Among B cell-intrinsic defects, at least five distinct groups are being recognized, namely defects in the B cell development, migration, activation, class switch recombination (CSR), and survival. Despite this classification, many of the PADs remain without a clear etiology [4].

The most prevalent of the PAD diagnoses is common variable immunodeficiency (CVID), an antibody deficiency caused by B cell dysfunction. The etiology of CVID seems to have a complex genetic cause, although few monogenetic causes may explain a CVID-like phenotype (e.g., *TACI*, *ICOS*, *CD19*, *CD20*, and *BAFFR*) [4, 5].

Twenty-fold less common than CVID are the so-called hyper-IgM syndromes (HIGM) or class switch recombination (CSR) defects, which are characterized by a low-to-normal or elevated serum IgM in the absence of any other immunoglobulin (Ig) isotypes in the serum. A number of unique gene defects (e.g., in *CD40L*, *CD40*, *AICDA*, *UNG*, and *IKBKG*) have been identified that lead to a defect in the CSR mechanism required for the transition of the initial low-affinity IgM to an Ig isotype of increased affinity [6, 7].

Defects in CD40L (TNFSR5 or CD154) encoded by the X-linked *CD40L* gene represented the first reported CSR defect. Identical abnormalities, both clinically as well as immunologically, have more recently been identified in the autosomal-recessive syndrome of mutations in the gene for its counter-receptor CD40 on B cells. In both syndromes, the lack of functional expression of the surface molecule, leads to the failure of a proper B cell-T cell signaling contact at the interphase of germinal centers where a subset of primary activated lymphocytes, i.e., the T follicular helper cells (Tfh) and naïve B cells meet in the lymph nodes, mucosal lymphoid tissues, or marginal zones of white pulp in the spleen [8]. After successful interaction between antigen-specific Tfh cells and B cells, the B cells start to proliferate and initiate a cell-intrinsic process of Ig affinity maturation by class switch and hypermutation, in which B cell-specific enzymes such as activation-induced cytidine deaminase or AID (encoded by *AICDA*) and uracil N-glycosylase activity (encoded by *UNG*) are involved [9–11].

Within the diagnosis and definitions of CVID, there are many overlapping features with CSR-defects. This is also recognized in the list of clinical criteria provided by the European Society for Immunodeficiencies (ESID). In CVID, the initial steps of B cell development in the bone marrow are supposed to have normally proceeded. In most patients, there are mature B cells circulating but in vivo antibody production fails. Many studies have tried to classify these unidentified defects in different subtypes of CVID using phenotyping and a description of the B cell recombination history [12–17].

We have previously used a comprehensive culture system to characterize CVID in functional terms by the capacity of the patient’s B cells to proliferate and differentiate into Ig-secreting plasmablasts (PBs) [15]. In this way, we have identified a subgroup of CVID patients, which was highly reminiscent of CSR-like B cell defects. Since this subgroup of CVID patients has not been well described, we have further characterized their B cell functions in greater detail as a separate group and compared the results of these CVID patients to those of patients with genetically well-defined CSR-defects.

## Materials and Methods

### Samples

This study was approved by the Medical Ethics Committee of the Academic Medical Center (Medisch Ethische Toetsingscommissie AMC) in Amsterdam, and was performed in accordance with the Declaration of Helsinki.

Heparinized peripheral blood samples from patients suspected or diagnosed with CVID were collected for routine diagnostics. Cord blood samples from healthy term-newborn donors were collected by nurses from the maternity ward with parental consent, all samples were collected anonymously for the authors and none of the authors had access to clinical data, samples were used in accordance with the Dutch law regarding the use of discarded material for research purposes. Healthy adult control samples (*n* = 20) were available from buffy coats obtained from Sanquin blood bank Amsterdam, all samples were acquired anonymously. None of the authors had access to clinical data from any control samples. PBMCs were isolated using standard density gradient centrifugation techniques using Lymphoprep (Nycomed, Oslo, Norway) and stored in liquid nitrogen until use. The CSR-deficient patients were identified via clinical presentation and corresponding diagnosis by gene analysis (of CD40L, CD40, AID, or UNG) by classical Sanger sequencing, all CVID patients were negative for these defects.

### Flow Cytometry

PBMCs were resuspended in phosphate-buffered saline (PBS), containing 0.5 % (*w*/*v*) BSA and 0.01 % sodium azide. PBMCs were incubated with saturating concentrations of fluorescently labeled conjugated monoclonal antibodies (MoAbs). Analysis of cells was performed using a FACSCanto-II flowcytometer and FlowJo software. The following directly conjugated MoAbs were used for flow cytometry: CD19-PerCP-Cy 5.5, CD19 Alexa-700, CD20-APC, CD20-PerCP-Cy 5.5, CD38 PE-Cy7, CD138 APC, IgG-PE, IgD-PE, and IgA-PE from BD-biosciences (San Jose, USA), CD27-FITC from Sanquin (Amsterdam, the Netherlands), IgM-PE from ITK-diagnostics (Uithoorn, the Netherlands).

### B Cell Activation In Vitro

PBMCs were resuspended in PBS at a concentration of 5–10 × 10^6^ cells/ml and labeled with 0.5 μM carboxyfluorescein succinimidyl ester (CFSE) (Molecular Probes) in PBS for 10 min at 37 °C under constant agitation. Cells were washed and subsequently resuspended in IMDM supplemented with 10 % fetal calf serum (BioWhittaker), antibiotics, and 3.57 × 10–4 %(*v*/*v*) β-mercapto-ethanol (Merck). Labeled PBMCs containing a fixed number of B cells (1 × 10^5^ per well) were cultured in 48-well flat-bottomed plates for 6 days at 37 °C and stimulated with saturating amounts of 5 μg/ml anti-IgM mAb (clone MH15; Sanquin), 1:500 anti-CD40 mAb ascites (clone 14G7; Sanquin), 20 ng/ml IL-21 (Invitrogen), or 1 μg/ml CpG oligodeoxynucleotide 2006 (Invivogen), with 100 U/ml IL-2 (R&D Systems). Proliferation of the B cells was assessed by measuring CFSE dilution by flow cytometry.

### IgG and IgM ELISA

Supernatants were tested for secreted IgM and IgG with an in-house ELISA using polyclonal rabbit anti-human IgG and IgM reagents and a serum protein calibrator all from Dako (Heverlee, Belgium), as described before [15]

### Statistics

Differences between immunoglobulin levels were calculated by two-sided, two-tailed Student’s *t* test. For correlations, the Spearman nonparametric correlation test was used. *P* < 0.05 was considered statistically significant.

## Results

### Patient Selection

Using our previously described experimental conditions for B cell activation [15], we have to date functionally screened over 40 PAD patients who were suspected or diagnosed with CVID by their clinicians because of their clinical presentation, low serum IgG and IgA, and a lack of humoral response to polysaccharides at presentation.

With this screening assay, we selected a small series of patients who were considered CSR-like CVID cases because of an increased IgM in their serum at diagnosis (more than 2SD above the cutoff for normal values measured at least two times apart) and/or documented “IgM-only” immunoglobulin isotype production in vitro (Tables 1 and 2).

**Table 1:** Clinical characteristics for CSR-like CVID patients at presentation

Patient	#1	#2	#3	#4	#5
Year of birth	1989	1939	1958	1965	1944
Gender	Male	Male	Male	Female	Female
Age at diagnosis	18	20	14	41	60
Clinical presentation					
	Appendicitis	Rec. lung infections	Bronchiectasis	Bronchiectasis	Rec. lung infections
	Rec. fever	Tonsillitis	Deafness	ITP	Bronchiectasis
	Pneumonia		Mild mental retardation	Splenomegaly	
	Sinusitis		Dental caries		
Gene analysis					
CD40L	neg	neg	neg	neg	neg
CD40	neg	neg	neg	neg	neg
UNG	neg	neg	neg	neg	neg
AICDA	neg	neg	neg	neg	neg
PI3Kdelta	neg	neg	neg	neg	neg
Immunoglobulins at presentation (g/L)					
IgG	<0.3	<0.1	1.2	2.5	<0.3
IgM	<0.03	0.46	7.05	5.27	2.5
IgA	<0.04	<0.05	<0.22	<0.22	<0.04
IgE	<2	ND	<2	<2	ND
B cell subsets at presentation					
IgD+CD27− (naive)	80.1	55.4	74	92	95.9
IgD+CD27+ (non-switched memory)	12.2	42.8	18.6	5	1.3
IgD−CD27+ (switched-memory)	4.2	1	0	0.7	0.2
IgD−CD27− (double negative)	3.5	0.8	7.4	2.3	2.6

Year of birth, patient gender, age at diagnosis, clinical presentation, mutations (*neg* tested negative), immunoglobulin levels at presentation, and B cell subset at presentation is shown

*ND* not done, *HUS* Hemolytic-uremic syndrome, *ITP* Idiopathic thrombocytopenic purpura

**Table 2:** Clinical characteristics for known CSR patients at presentation

Patient	CD40L #1	CD40L #2	AID #1	AID #2	UNG
Year of birth	1985	1980	1975	1972	1994
Gender	Male	Male	Female	Male	Male
Age at diagnosis	3	2	20	6	3
Clinical presentation					
	Bronchiectasis	Rec. neutropenia	Vasculitis	Rec. lung infections	Rec. lung infections
	Sinusitis	HUS	Rec. breast abces	Bronchiectasis	Rec. Otitis
	Cold sores		Meningococcal meningitis		Failure to thrive
	Urticaria		Cutaneous lupus		
Gene analysis					
CD40L	c.464T>C, (p. Leu155Pro)	c.435delC	–	–	–
UNG	–	–	–	–	c.630_632delCCT
AICDA	–	–	c.156+1T>G	c.317T>C (p. L106P)	–
Immunoglobulins at presentation (g/L)					
IgG	1.5	<0.1	<0.1	<0.1	<0.1
IgM	7.5	3.5	9.0	ND	0.5
IgA	<0.22	<0.1	<0.1	ND	<0.1
IgE	<2	ND	ND	ND	ND
B cell subsets at presentation					
IgD+CD27− (naive)	92	ND	ND	ND	68.6
IgD+CD27+ (non-switched memory)	7	ND	ND	ND	27.4
IgD−CD27+ (switched-memory)	0	ND	ND	ND	2.4
IgD−CD27− (double negative)	1	ND	ND	ND	1.6

When categorized according to the Freiburg, Paris, and EUROclass classification [17], these CSR-like CVID patients fitted in the B+ CVID patient category with a variable number of memory B cells but without a significant expansion of transitional B cells (Table 3).

**Table 3:** B cell subset characteristics at time of analysis for CSR patients and CSR-like CVID patients

B cell subsets					
Identifier	%Naive	%Non-Switched	%Memory	%IgD−CD27−	%Transitional
Adult control	(45–85)	(5–25)	(9.0–35)	(1.3–4.3)	(1.7–3.7)
Cord blood	(97–100)	(0.0–1.6)	(0.0–0.2)	(0.1–1.2)	(5.0–12.0)
CD40L	94.3	4.0	0.6	0.9	7.7
AID	71.9	19.6	5.1	3.2	4.6
UNG	83.8	13.0	0.4	2.6	1.56
Patient #1	80.1	12.2	4.2	3.5	1.2
Patient #2	54.9	42.5	0.9	1.5	0.1
Patient #3	70.3	21.9	5.6	2.2	1.6
Patient #4	92.6	1.9	0.9	4.6	2.3
Patient #5	92.5	4.5	1.4	1.6	3.2

The range for all different peripheral B cell subsets of adult control (*N* = 25) and cord blood (*N* = 15) samples, with average percentages (mean) of the different B cell subsets for the CSR patients and CSR-like CVID patients (from *N* = 3–5 experiments), gated for CD19^+^ cells is shown

*ND* not done

These selected CVID patients had normal T cell numbers and function upon T cell activation toward anti-CD3, anti-CD3/anti-CD28, IL7, or IL15, as indicated in proliferation assays as described previously (data not shown).

### Normal Peripheral Blood B Cell Phenotypes

**Fig. 1:**
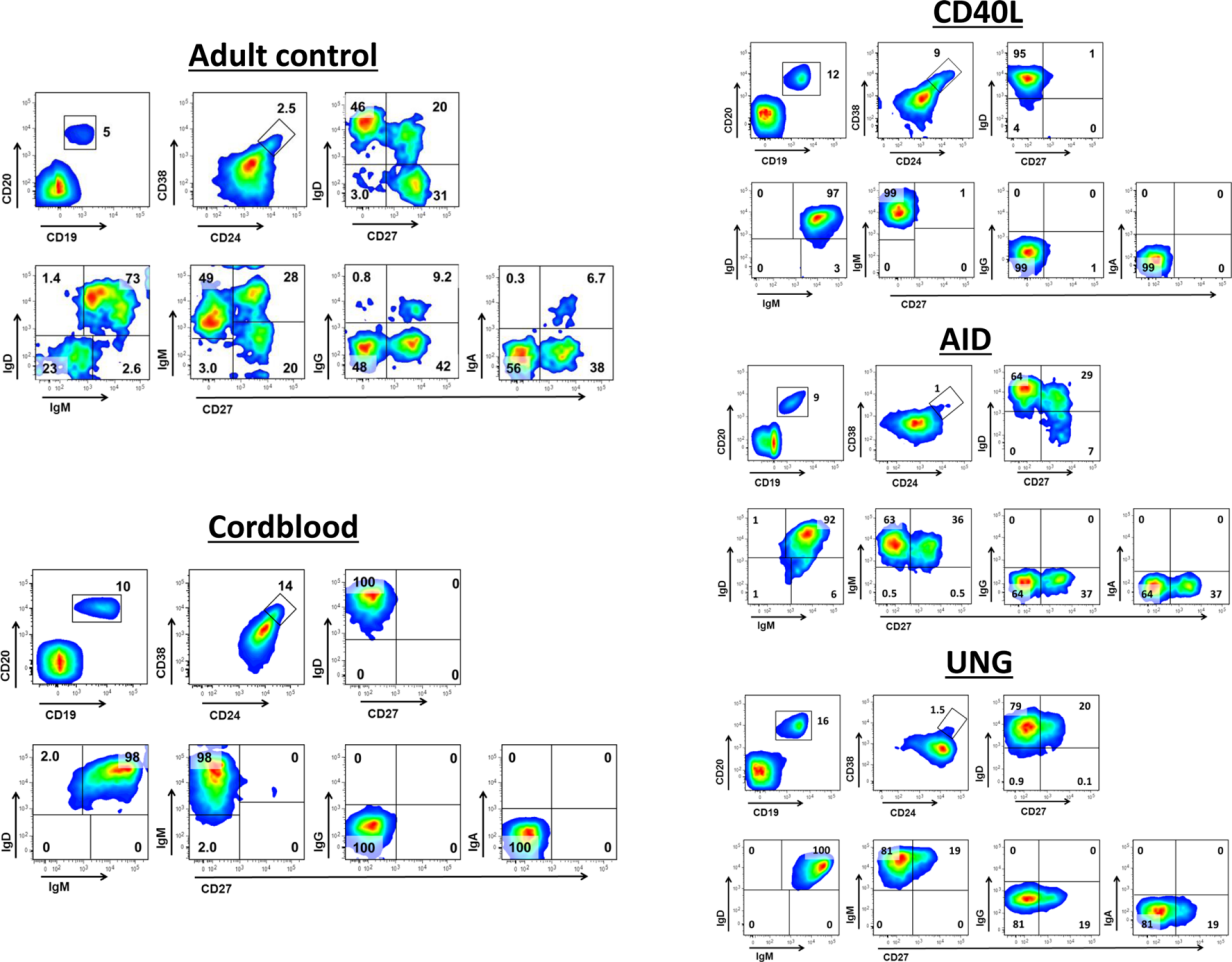
Representative figures of the phenotype of circulating B cells from healthy adult controls, healthy cord bloods, and CD40L-, AID-, and UNG-deficient patients. B cell subsets of representative blood samples from healthy adult and cord blood samples, as well as from genotyped CD40L-, AID-, and UNG-deficient patients. *Numbers* indicate mean percentages of multiple experiments in the corresponding quadrant. Healthy adult controls (*N* = 20), healthy cord bloods (*N* = 15), and CD40L-, AID-, and UNG-deficient patients (*N* = 2–5)

Within the B cell compartment (CD20^+^CD19^+^), various B cell subsets are routinely distinguished, i.e., transitional (CD38^high^CD24^high^), naïve (sIgD^+^CD27^−^), non-switched (sIgD^+^CD27^+^), and switched memory (sIgD^−^CD27^+^) B cells. During childhood, the human B cell compartment changes from a completely naive to a more differentiated phenotype as a consequence of the expansion of CD27^+^ memory B cells. Within the CD27^+^ memory B cell compartment, surface immunoglobulin receptor expression can be used to further distinguish sIgM^+^, sIgG^+^, and sIgA^+^ memory B cells [18–20]. In the adult PBMC fractions, the B cell phenotype demonstrates the presence of a clear memory B cell compartment including sIgG^+^ and sIgA^+^ B cells, both of which are absent in cord blood PBMCs where all B cells are naïve (Fig. 1 and Supplementary Fig. Fig. 1).

### Patients with Classical CSR Defects Show Phenotypic Differences in Peripheral B Cells

Patients with genetically well-characterized CSR defects were included (Table 2) for comparison with the five CSR-like CVID patients mentioned earlier (Table 1). Next to cord-blood samples and healthy adult samples, we immunophenotyped the classical CSR cases to directly compare with the CSR-like CVID patients (Table 3). As reported before [8], the circulating B cells in patients with *CD40L* gene defects consisted of naïve B cells only and no memory B cells. These patients did have a slightly increased number of transitional B cells, similar to cord blood samples. On the other hand, patients who suffered from defects in *AICDA* showed normal numbers of non-switched B cells and even some memory sIgD^−^CD27^+^ B cells that had not undergone any class switching, i.e., these cells did not show any sIgG or sIgA expression and expressed sIgM only. Similar to patients with an *AICDA* gene defect, the individual that had been identified with an *UNG* gene defect [15], contained non-switched sIgM^+^ B cell population in the absence of sIgD^−^CD27^+^ B cells, indicating a lack of switched sIgG^+^ and sIgA^+^ memory B cells (Table 3).

### Plasmablast Formation Upon Activation of Healthy B Cells

The capacity of the B cells to proliferate and differentiate upon in vitro activation in a 6-day culture was tested with CpG in the presence of a small B cell activating dose of IL-2 (to which purified T cells do not show proliferation and cytokine induction and acts by direct B cell activation of the IL-2 receptor) [15, 21]. T cell-dependent B cell stimulation was mimicked by the combination of antibodies against sIgM to trigger the B cell antigen-receptor (BCR) on the majority of circulating B cells in the blood, together with costimulatory CD40 activation and Tfh cell-associated IL-21 (αIgM/αCD40/IL-21) [22]. To check for the T cell function and the indirect effects of T cell proliferation on subsequent B cell activation, we also stimulated the PBMCs with the combination of T cell-specific αCD3/αCD28 MoAbs, in which the common-gamma (CD132)-cytokine receptors play an essential role as we had previously described [18].

**Fig. 2:**
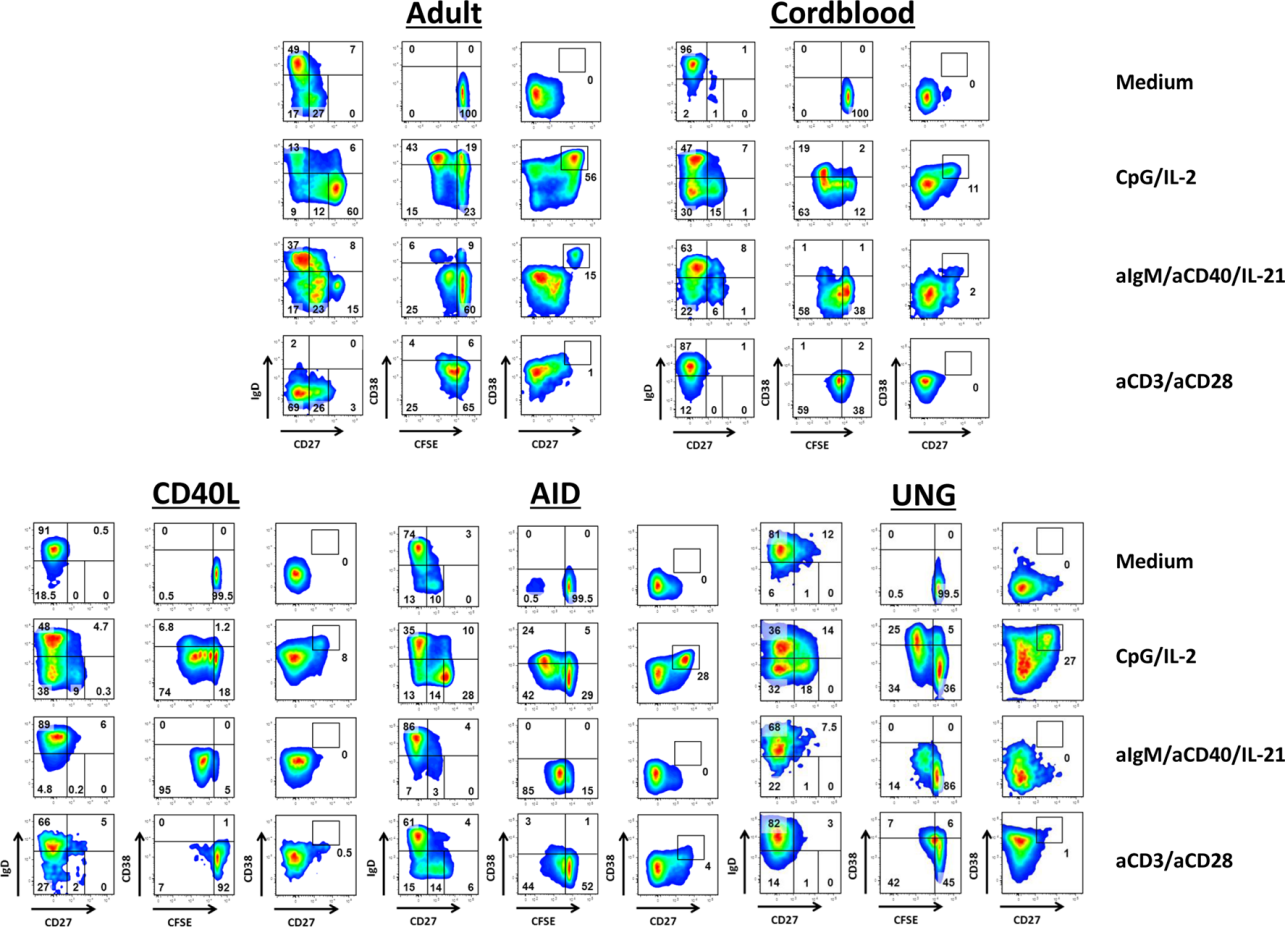
Proliferation and differentiation of B cells from CSR-deficient patients upon activation. The capacity of B cells from healthy adult controls, healthy cord bloods, and CD40L-, AID-, and UNG-deficient CSR patients to proliferate and differentiate in vitro were tested. CFSE-labeled PBMCs were cultured for 6 days, normalized for B cell numbers (1 × 10^5^ B cells/well). T cell-independent B cell activation was tested with CpG in the presence of IL-2. T cell-dependent B cell stimulation was mimicked by the combinations of αIgM/αCD40/IL-21. Effect of T cell stimulation was mimicked by αCD3/αCD28 stimulation, targeting T cells specifically. Representative FACS plots are shown of B cell subset distribution after 6 days of culture in the presence of the indicated stimuli. Gated on CD19^+^ lymphocytes to show CFSE dilution indicating proliferation after 6 days of culture, and to demonstrate the emergence of the subsets of Ig-producing B cells, i.e., plasmablasts and/or plasmacells (sIgD^−^/CD27^++^/CD38^++^)

In control experiments, we showed that upon activation, the adult B cells proliferated and differentiated into PBs (sIgD^−^CD27^++^CD38^++^) (Fig. 2 and Supplementary Fig. Fig. 2). Cord blood B cells showed similar responses but largely failed to differentiate into PBs after 6 days of stimulation. Both adult and cord blood B cells showed proliferation upon T cell-specific αCD3/αCD28 stimulation. The αCD3/αCD28 activation downregulated sIgD only on adult and not the cord blood B cells after 6 days of culture, but PBs expressing high levels of CD27 or CD38 did not develop under these conditions.

### Plasmablast Formation Upon CSR-Defective B Cell Activation

We subsequently analyzed the B cells of the known CSR defect patients. With regard to CD40L-deficient B cells, these cells proliferated normally upon CpG/IL-2 stimulation but B cells could hardly differentiate into CD27^++^CD38^++^ PBs. Stimulation with αIgM/αCD40/IL-21 induced vigorous B cell proliferation without any differentiation into PBs at all (Tables 4 and 5). Remarkably, the αCD3/αCD28-mediated T cell activation failed completely to induce any B cell proliferation in both CD40L-deficient patients. Identical responses were found in a CD40-deficient patient, demonstrating the importance of CD40L-CD40 interactions (data not shown).

**Table 4:** Proliferation, plasmablast differentiation and release of immunoglobulin after 6-day stimulation with CpG/IL-2 of CSR patients and CSR-like CVID patients

CpG/IL-2						
Identifier	Proliferation	CD27++	CD38++	CD20dull	IgG	IgM
Adult control	+	+	+	+	+	+
Cord blood	+	+/−	+/−	+/−	−	++
CD40L	+	+/−	+/−	−	−	+/−
AID	+	+	+	+	−	++
UNG	+	+/−	+	−	−	+
Patient #1	+	+	+	+	−	++
Patient #2	+	+/−	+	−	−	+
Patient #3	+	+	+	+	−	+/−
Patient #4	+	+/−	+/−	−	−	+/−
Patient #5	+/−	−	−	−	−	+/−

Proliferation (measured by CFSE dilution), differentiation into plasmablast (phenotypical markers (CD27^++^, CD38^++^, and CD20^dull^)) and release of immunoglobulin into the supernatant (by ELISA) after stimulation with CpG/IL-2 are scored in comparison with healthy adult controls: ++ (above controls), + (average), +/− (below controls) or – (absent). All data shown are gated on the CD19^+^ B cell population. Adult control (*N* = 25), cord blood (*N* = 15), CSR patients and CSR-like CVID patients (*N* = 3–5)

**Table 5:** Proliferation, plasmablast differentiation and release of immunoglobulin after 6-day stimulation with aCD40/IL-21 of CSR patients and CSR-like CVID patients

aCD40/IL-21						
Identifier	Proliferation	CD27++	CD38++	CD20dull	IgG	IgM
Adult control	+	+	+	+	+	−
Cord blood	+	+/−	+/−	+/−	−	−
CD40L	+	−	−	−	−	−
AID	+	−	−	−	−	+/−
UNG	+	−	−	−	−	−
Patient #1	+	−	−	−	−	+/−
Patient #2	+/−	−	−	−	−	−
Patient #3	+	−	−	−	−	ND
Patient #4	+	−	−	−	−	−
Patient #5	+/−	−	−	−	−	−

Proliferation (measured by CFSE dilution), differentiation into plasmablast (phenotypical markers (CD27^++^, CD38^++^ and CD20^dull^)) and release of immunoglobulin into the supernatant (by ELISA) after stimulation with aCD40/IL-21 are scored in comparison with healthy adult controls: ++ (above controls), + (average), +/− (below controls) or – (absent). All data shown are gated on the CD19^+^ B cell population. Adult control (*N* = 25), cord blood (*N* = 15), CSR patients and CSR-like CVID patients (*N* = 3–5)

Stimulation of the B cells of *AICDA*-mutated patients resulted in a different B cell signature in our B cell cultures. Both B cell proliferation and differentiation into PBs were observed upon activation with CpG/IL-2. In contrast, αIgM/αCD40/IL-21 stimulation induced B cell proliferation but no PB formation—similar to cord blood B cells and the CD40L- or CD40-deficient B cell cultures. With αCD3/αCD28 stimulation, AID-deficient B cells proliferated normally, but did not downregulate sIgD (in contrast to control adult B cells).

The reactivity of B cells of the *UNG*-mutated patient was an almost complete functional phenocopy of the *AICDA*-mutated patients. B cells from the *UNG*-mutated patient showed less proliferation when stimulated with αIgM/αCD40/IL-21, and showed no differentiation into PBs.

Thus, B cells from patients with CD40L or CD40 CSR defects showed an in vitro pattern of proliferation and differentiation responses different from AID or UNG-defective B cells (Tables 4 and 5).

### In Vitro Release of Immunoglobulins in Case of Known CSR Defects

**Fig. 3:**
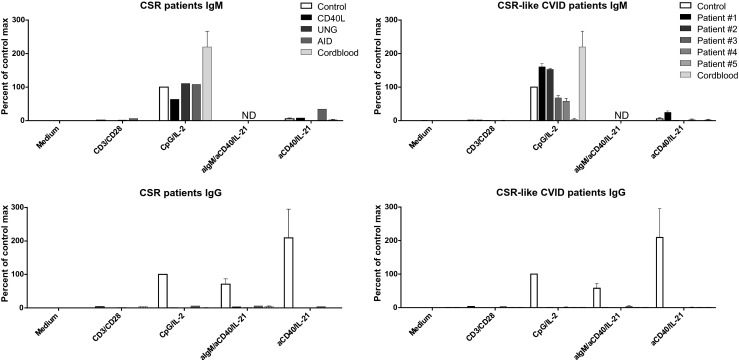
Production and release of immunoglobulins from CSR-deficient patients and CSR-like CVID patients upon B cell activation. IgM and IgG levels were measured in the supernatants by ELISA after 6 days of culture. Plotting multiple experiments per patient group (*N* = 2–5). Immunoglobulin production shown is normalized to that of the healthy control samples used in each separate experiment, production of Ig’s after stimulated with CpG/IL-2 is set at 100 %. Controls range from 2700 to 5000 pg/ml for IgG and 4000–9000 pg/ml for IgM. *ND* not done

Both IgG and IgM were measured in the supernatants of the 6-day cultures (Fig. 3). Stimulation with CpG/IL-2 resulted in the production of IgG and IgM by adult B cells, but only IgM was produced by cord blood B cells.

Since stimulation of the cell cultures with αIgM/αCD40/IL-21 makes the detection of IgM unreliable because of the addition of αIgM, we changed to stimulation with αCD40/IL-21 alone, resulting in similar B cell proliferation and PB differentiation in adult control cells (data not shown). Both αIgM/αCD40/IL-21 and αCD40/IL-21 stimulation resulted in the release of IgG in control PBMC cultures but—as expected—not in cord blood-derived samples. Noticeably, we did not detect any IgM release with αCD40/IL-21 stimulation. Although strong proliferation was induced upon αCD3/αCD28 stimulation, neither adult nor cord blood B cells produced IgG or IgM under these conditions.

As expected, none of the B cell cultures performed with PBMCs from CSR-deficient patients produced any IgG in vitro for all the conditions tested. In contrast, CpG/IL-2 stimulation resulted in massive IgM release in 6-day cultures by the cells from patients with mutations in *UNG* or *AICDA*, and to a lesser extent by those with the *CD40L* mutations (Table 4). B cells of patients with an *AICDA* defect were unique in their capacity to produce IgM when stimulated with αCD40/IL-21, in contrast to B cells from other genetically characterized CSR defects or from any of the normal controls tested thus far (over 100 individuals to date).

### Diagnostic Categorization of Unidentified CSR-like CVID

The patients had normal or increased serum IgM in vivo, apart from patient #1, who had no detectable IgM in serum. All patients had circulating B cells that produced relatively large amounts of IgM without any IgG and IgA upon in vitro activation.

Their B cell functionality was compared to the B cell cultures of our patients with known CSR defects (Fig. 3, Tables 4 and 5). One of the selected CSR-like CVID patients had a child with exactly the same immunophenotype and clinical diagnostic features (patient #2). In all of these patients, classical CSR defects were excluded, i.e., phenotypic or genetic defects in *CD40L*, *CD40*, *AICDA*, *UNG*, or the recently identified PI3K defects, established in Activated PI3K-p110delta syndrome, types 1 and 2 (APDS-1 and APDS-2, caused by a number of gain-of-function mutations in *PIK3CD* and *PIK3R1*, respectively) [23–25].

**Fig. 4:**
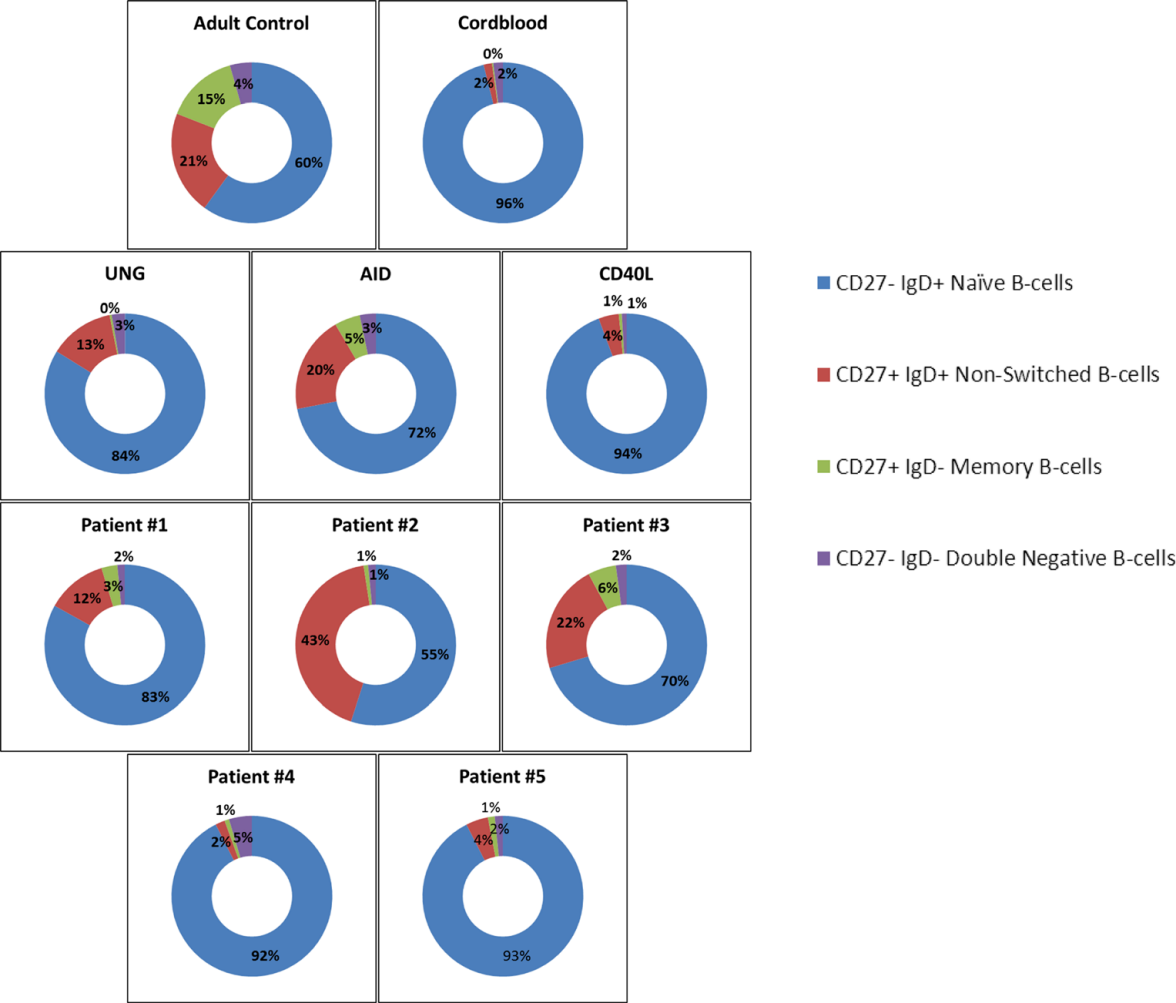
Representative diagrams of the circulating B cell phenotype at time of analysis. B cell subsets of representative blood samples from a healthy adult control, healthy cord blood, CD40L-, AID-, UNG-deficient CSR patients and five CSR-like CVID patients. *Numbers* indicate mean percentages of multiple experiments in the corresponding quadrant. Healthy adult controls (*N* = 20), healthy cord bloods (*N* = 15), CD40L-, AID- and UNG-deficient patients (*N* = 2–5), CSR-like CVID patients (*N* = 2–5)

The immunophenotype of the peripheral circulating B cells of these CVID patients was compared to the B cell phenotype of the patients with classical CSR defects (Fig. 4). Three of our patients (patients #1, #2, and #3) looked very similar to those with an *AICDA* or *UNG* defect, i.e., their B cell compartment comprised of naïve and non-switched B cells only, with very few if any switched memory B cells. The other two patients (patients #4 and #5) resembled the CD40L-deficient patients, i.e., completely naïve in their B cell compartment without any circulating non-switched and switched CD27^+^ memory B cells.

Also, after stimulation with CpG/IL-2 and αCD40/IL-21 (Table 4 and 5), our results showed that patients #1, #2, and #3 mostly resembled the B cell-intrinsic AID/UNG-deficient patients, i.e., the B cells proliferated under all conditions of B cell activation tested and induced proper B cell differentiation and IgM production in vitro upon CpG/IL-2 activation.

On the other hand, patients #4 and #5 behaved differently. Patient #4 again resembled the CD40L-deficient patients with a normal proliferation upon CpG/IL-2 stimulation and αIgM/αCD40/IL-21 but without any PB differentiation. However, this patient’s B cells demonstrated a completely normal B cell proliferation upon T cell activation with αCD3/αCD28 which was absent with CD40L- or CD40-deficient B cells (Fig. 2 and data not shown).

In contrast to the other CSR-like cases as well as the classical CSR patients, the B cells of patient #5, who presented with an elevated serum IgM upon diagnosis, repeatedly showed a lack of proliferation upon any kind of B cell stimulation and failed to differentiate to PBs with almost no IgM production (6–10 % of control values) and IgG (or IgA) production, hence not resembling any of the tested CSR patients.

## Discussion and Conclusions

We describe a subgroup of patients with CVID identified by a distinct failure of in vitro activation of the B cells into IgG/IgA-secreting PBs in the presence of a normal or increased IgM secretion. This defect in Ig class switching is not based on known CSR gene defects. Clinically, these patients were mainly characterized by recurrent infections.

When combining our previously described functional B cell classification with the results of our cultures in a series of patients who had been genetically characterized by one of the currently known CSR defects, we can add an additional layer to identify the possible B cell defects among patients hitherto diagnosed with CVID.

Having determined the B cell reactivity of the most common CSR defects in our culture system by analysis of proliferation, differentiation, and PB formation, we noticed a striking similarity between a subgroup of CVID patients and patients with genetically well-characterized CSR defects. In the subgroup of CSR-like CVID patients, a major T cell defect was excluded by the normal T cell counts and subsets (naïve, memory, memory-effector T cell subsets according to standard CD45RA/CD27 staining [26]) (Supplementary table 1), as well as their proliferative capacity upon various stimuli including αCD3 and αCD3/αCD28 stimulation (data not shown).

The functional data from the CSR-like CVID patients reported here suggest that the failure of B cells to proliferate and differentiate upon stimulation may be localized at distinct stages of differentiation. One patient identified in this small series of CVID cases best fitted the category of a very early B cell activation defect (patient #5), because both B cell proliferation and differentiation were clearly defective.

The signal to recombine to a particular switch region comes from the cell surface. Cytokines such as interleukin-21 or IL-4, and appropriate co-stimulation with CD40L induce the production of so-called sterile transcripts from promoters that are upstream of the targeted switch regions. A recent study has highlighted the mechanism of how such transcription through the immunoglobulin switch region produces a non-coding RNA that guides the enzyme AID to DNA in a sequence-specific manner to promote antibody CSR [27].

As had been shown before [28], in vitro stimulation of B cells that lacked AID resulted in the production of sterile transcripts without CSR. While this recent study from Zheng et al. [27] also suggest that the switch RNA debranching enzyme DBR1 influences CSR, the relative difficulty of performing these experiments in these patient materials did not permit us to further investigate at which steps these CSR-defective CVID patients fail in the CSR process. Noticeably, the B cell cultures of our patients reacted differently to stimulation, suggesting that the CSR failure was not identical. It would be interesting to investigate whether sterile transcripts, SHM, or AICDA expression are normal in these patient B cells or whether signaling or metabolic processes otherwise determine their failure in CSR activity.

In functional terms, the CD40L-deficient patients and patient #4 were most similar apart from the defective B cell proliferation after αCD3/αCD28-mediated T cell activation when CD40L is deficient. We have observed a similar lack of αCD3/αCD28-mediated B cell response in leaky X-CID patients with hypomorphic *IL2RG* gene mutations encoding the common gamma chain [18, 29]. We therefore conclude that CD40L-CD40 interaction together with the cytokine production by T cells—and in particular, IL-21 as suggested from blocking experiments (data not shown)—are responsible for the B cell proliferation by αCD3/αCD28-mediated T cell activation [8, 30]. Patient #4 can be clearly distinguished from these activation defects.

Finally, the B cells of CSR-like patients #1, #2, and #3 showed clear overlap with the B cell-intrinsic CSR defects of AID- and UNG-deficient patients, where normal B cell proliferation and differentiation into PBs was found, as well as massive in vitro production of IgM without any IgG or IgA. Absence of sIgG and sIgA on the B cells ex vivo supports the lack of Ig-isotype switched memory cells. The lack of IgG/A production with normal in vitro proliferation and differentiation defects resembles the cord blood phenotype; however, cord blood samples have a completely naïve B cell subset whereas AID- and UNG-deficient patients have clear population of circulating sIgD^+^CD27^+^ B cells. Another interesting finding is that patient #1 as well as our AID-deficient patients produce a moderate amount of IgM when stimulated with αC40/IL-21, this in contrast to all other samples. Potentially showing the failure in class switching, or a compensatory mechanism for the lack of IgG production.

Our approach to categorize CVID-patients according to functional B cell activation and differentiation may give additional information about the underlying signaling pathways contributing to the observed B cell defects in CVID.

Although a single gene defect may still underlie this distinct CSR-like CVID subgroup, our data already demonstrate variability in these CSR-like B cell defects, making a monogenetic cause rather unlikely. Even within the same PID subcategory, defects can lead to variable phenotypes, which could be the result of genotype (hypomorphic mutations), the microbial exposure, and/or the complex nature of the gene defect causing a disbalance or loss of strength of multiple activating signals. For instance, data from the recently described defects in the mTOR-AKT-PI3K pathway in humans and mice already hints to such variability in the outcome of functional B cell development [31–34]. In APDS, the observed dysglobulinemia may encompass a slight increase of IgM and decrease in IgG or IgG-subclass levels. Also, mutations in the CTLA4 gene have recently been shown to cause CVID with highly variable and incomplete penetrance [35, 36].

For our subgroup of functionally characterized CSR-like CVID patients, whole genome sequencing has been undertaken. Known PID genes according to the IUIS list of approved PIDs [37] did not show any defect in the CSR-like CVID cases to date. RNA expression arrays may for that reason be informative to define this subgroup in more detail to further explore whether a disbalance in B cell-activating signals or additional genes involved in CSR, may be involved.

In sum, our culture system to functionally test CVID patients by determining the capacity of peripheral blood B cells to proliferate and differentiate into activated Ig-secreting PBs may be useful to characterize hitherto undescribed functional PAD subgroups. Our data on a distinct CVID subgroup indicates the presence of B cell defects comparable with known CSR patients. Their recognition as a subgroup in functional terms may help to identify the underlying defects in B cell activation.

## Electronic Supplementary Material

Below is the link to the electronic supplementary material.

Supplementary Fig. 1 In-depth phenotype of circulating B cells from healthy adult controls, healthy cord bloods, CD40L-, AID-, and UNG-deficient patients. B cell subsets from healthy adult and cord blood samples, as well as from genotyped CD40L-, AID- and UNG-deficient CSR patients. Quantification of B cell subsets with mean of healthy adult controls (*N* = 20); healthy cord bloods (*N* = 5–10); and CD40L-, AID-, and UNG-deficient patients (*N* = 1–3). CD27/IgD, CD27/IgM, CD27/IgG and CD27/IgA subsets is shown. (GIF 69 kb)

High Resolution Image (TIF 377 kb)

Supplementary Fig. 2 Differentiation of B cells from healthy adult, healthy cord blood, and know CSR-deficient patients upon activation. The capacity of B cells from healthy adult controls, healthy cord bloods, and CD40L-, AID-, and UNG-deficient patients to proliferate and differentiate in vitro were tested. CFSE-labeled PBMCs were cultured for 6 days, normalized for B cell numbers (1 × 10^5^ B cells/well). T cell-independent B cell activation was tested with CpG in the presence of IL-2. T cell-dependent B cell stimulation was mimicked by the combinations of αIgM/αCD40/IL-21. Effect of T cell stimulation was mimicked by αCD3/αCD28 stimulation, targeting T cells specifically. Quantification of B cell subsets distribution after 6 days of culture in the presence of the indicated stimuli. Gated on CD19^+^ lymphocytes to show CFSE dilution indicating proliferation after 6 days of culture, and to demonstrate the emergence of the subsets of Ig-producing B cells, i.e., plasmablasts (sIgD^−^/CD27^++^/CD38^++^). Healthy adult controls (*N* = 20), healthy cord bloods (*N* = 5–10). For the CD40L-, AID-, and UNG-deficient patients, we grouped multiple experiments (*N* = 3–5 per patient). This shows clear failure of CSR patients B cells to differentiate into plasmablasts. (GIF 33 kb)

High Resolution Image (TIF 242 kb)

Supplementary Table 1 T cell subsets of CSR-like CVID patients. (DOC 42 kb)
