# Supplementary figures and images for: Phenotypic and Functional Comparison of Class Switch Recombination Deficiencies with a Subgroup of Common Variable Immunodeficiencies
Source: J Clin Immunol. 2016 Aug 2;36(7):656–66. doi: 10.1007/s10875-016-0321-2 (PMC5018261; doi:10.1007/s10875-016-0321-2)

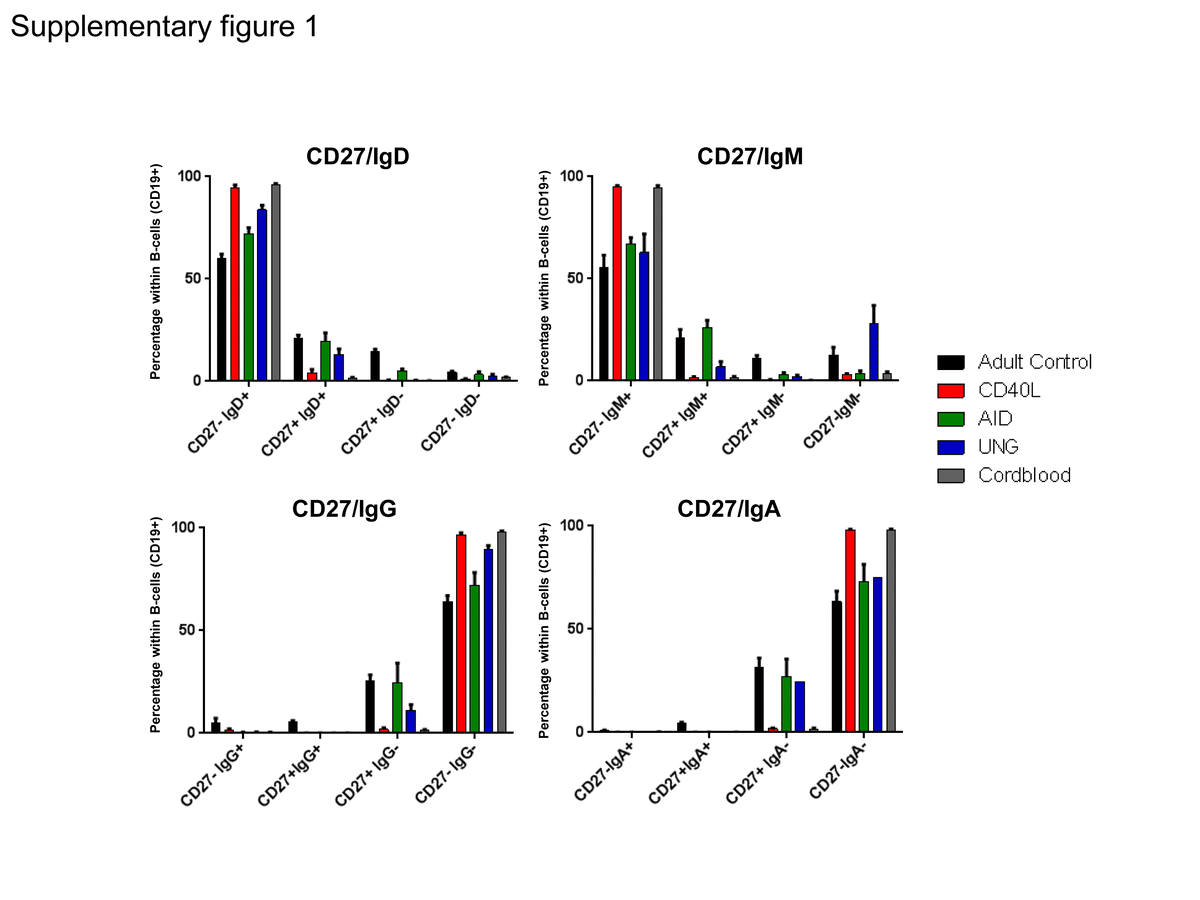

Supplement: Supplementary file 1 — In-depth phenotype of circulating B cells from healthy adult controls, healthy cord bloods, CD40L-, AID-, and UNG-deficient patients. B cell subsets from healthy adult and cord blood samples, as well as from genotyped CD40L-, AID- and UNG-deficient CSR patients. Quantification of B cell subsets with mean of healthy adult controls (N = 20); healthy cord bloods (N = 5–10); and CD40L-, AID-, and UNG-deficient patients (N = 1–3). CD27/IgD, CD27/IgM, CD27/IgG and CD27/IgA subsets is shown. (GIF 69 kb) [file 10875_2016_321_Fig5_ESM.gif]

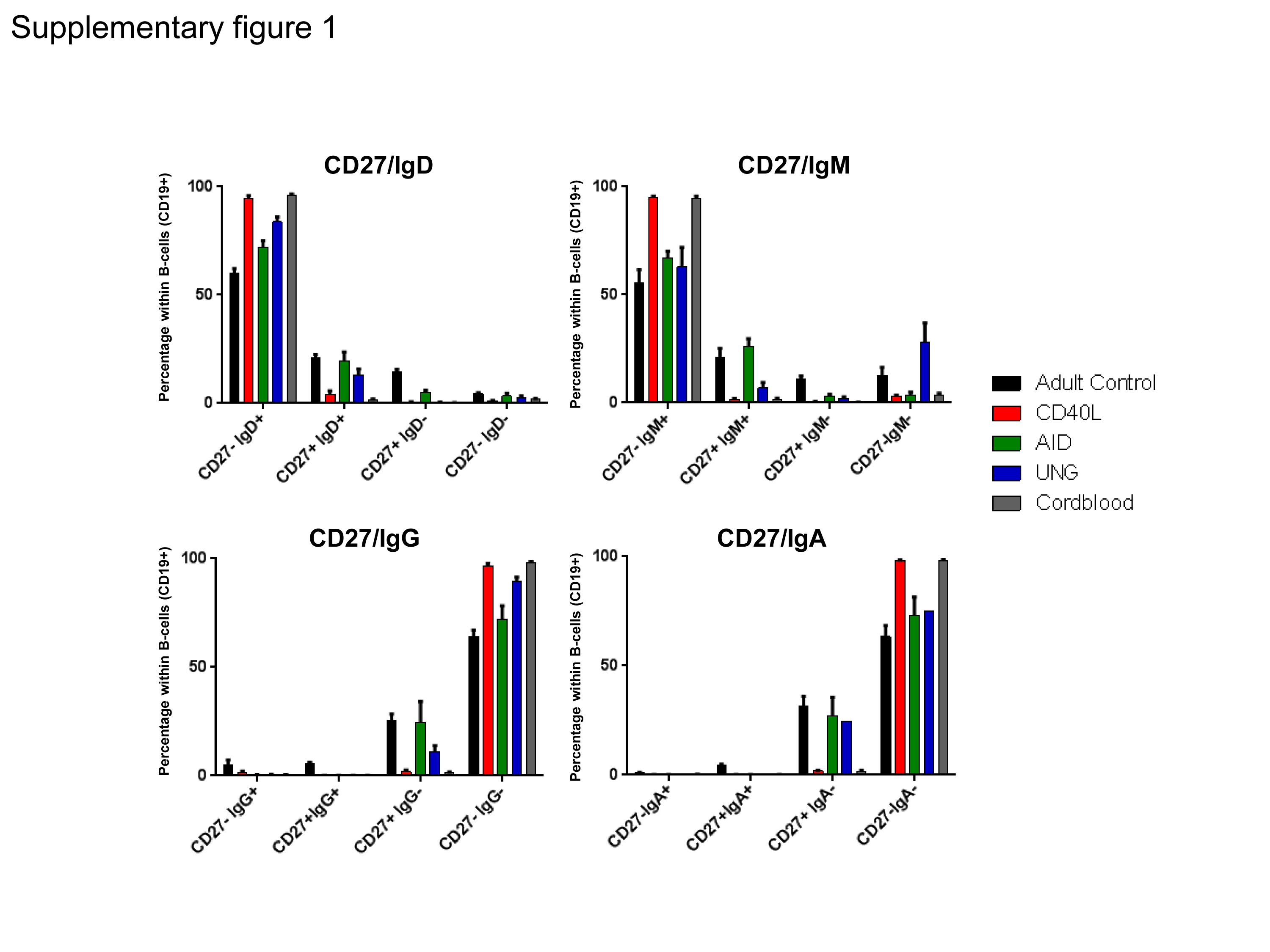

Supplement: Supplementary file 2 — High Resolution Image (TIF 377 kb) [file 10875_2016_321_MOESM1_ESM.tif]

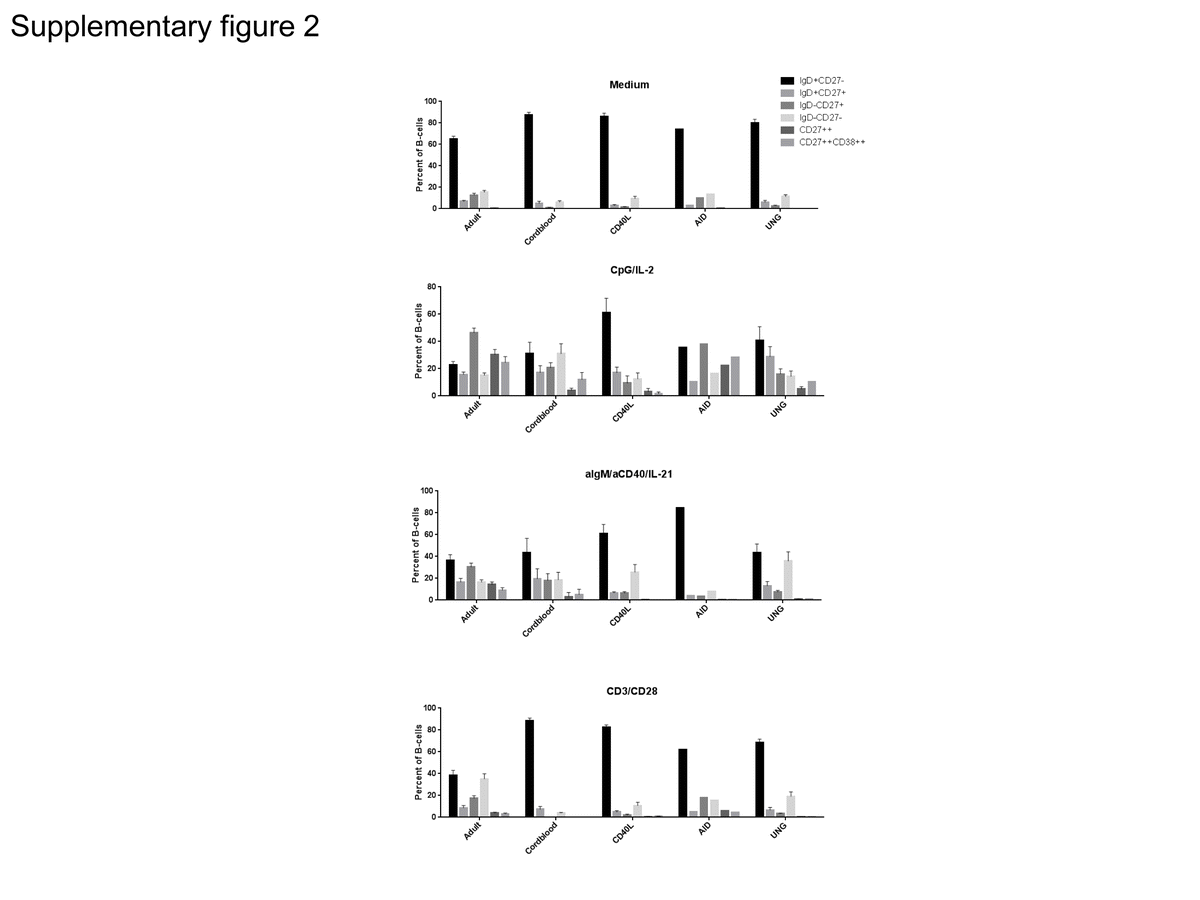

Supplement: Supplementary file 3 — Differentiation of B cells from healthy adult, healthy cord blood, and know CSR-deficient patients upon activation. The capacity of B cells from healthy adult controls, healthy cord bloods, and CD40L-, AID-, and UNG-deficient patients to proliferate and differentiate in vitro were tested. CFSE-labeled PBMCs were cultured for 6 days, normalized for B cell numbers (1 × 10^5^ B cells/well). T cell-independent B cell activation was tested with CpG in the presence of IL-2. T cell-dependent B cell stimulation was mimicked by the combinations of αIgM/αCD40/IL-21. Effect of T cell stimulation was mimicked by αCD3/αCD28 stimulation, targeting T cells specifically. Quantification of B cell subsets distribution after 6 days of culture in the presence of the indicated stimuli. Gated on CD19+ lymphocytes to show CFSE dilution indicating proliferation after 6 days of culture, and to demonstrate the emergence of the subsets of Ig-producing B cells, i.e., plasmablasts (sIgD−/CD27++/CD38++). Healthy adult controls (N = 20), healthy cord bloods (N = 5–10). For the CD40L-, AID-, and UNG-deficient patients, we grouped multiple experiments (N = 3–5 per patient). This shows clear failure of CSR patients B cells to differentiate into plasmablasts. (GIF 33 kb) [file 10875_2016_321_Fig6_ESM.gif]

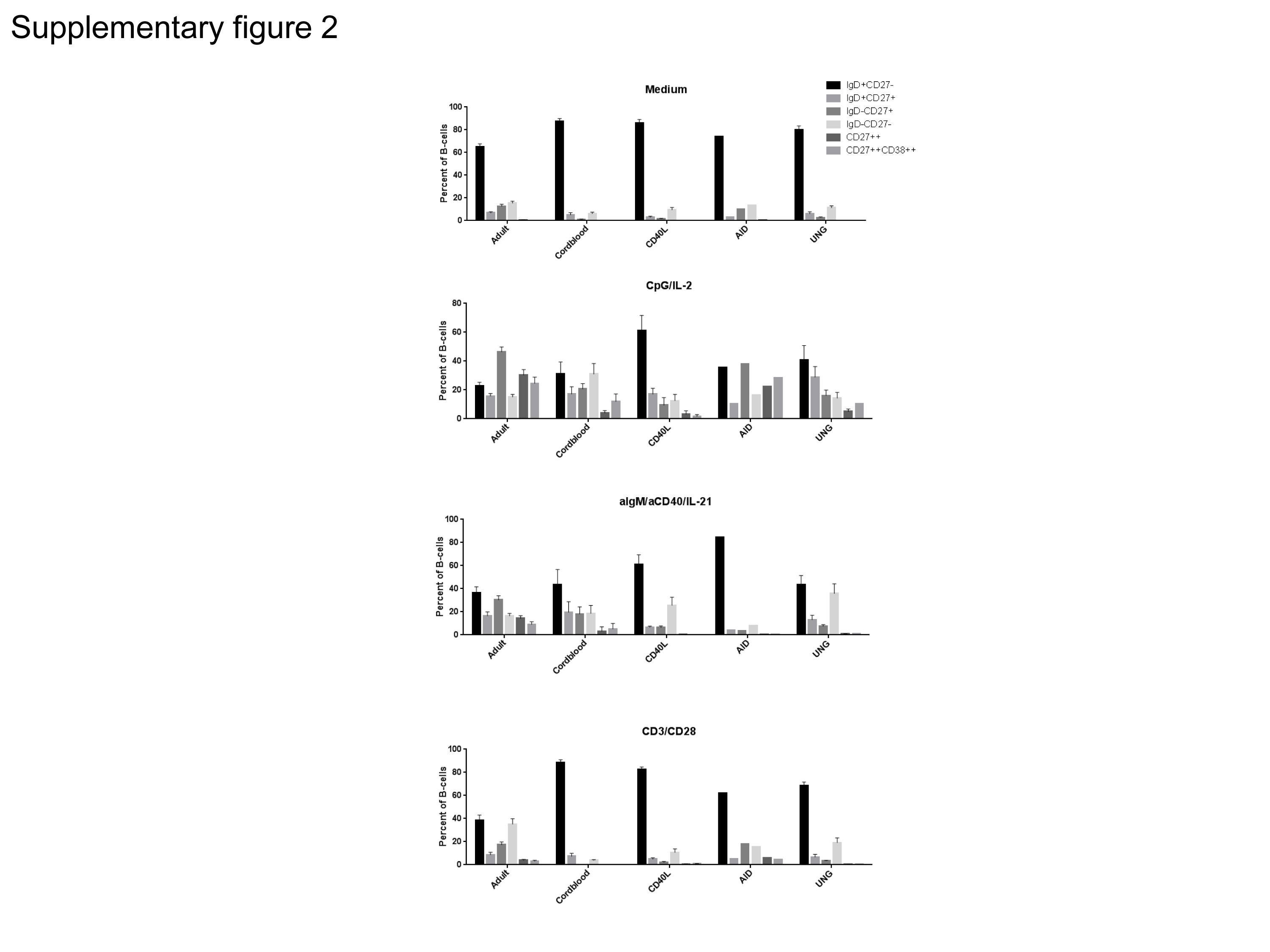

Supplement: Supplementary file 4 — High Resolution Image (TIF 242 kb) [file 10875_2016_321_MOESM2_ESM.tif]
