# Supplementary material for: Phenotypic and Functional Comparison of Class Switch Recombination Deficiencies with a Subgroup of Common Variable Immunodeficiencies
Source: J Clin Immunol. 2016 Aug 2;36(7):656–66. doi: 10.1007/s10875-016-0321-2 (PMC5018261; doi:10.1007/s10875-016-0321-2)
Supplement: Supplementary file 5 — T cell subsets of CSR-like CVID patients. (DOC 42 kb) [file 10875_2016_321_MOESM3_ESM.doc]

**Supplementary Table 1**

Supplementary Table 1: **T cell subsets of CSR-like CVID patients.**

T cell subsets of CSR-like CVID patients. Showing absolute counts and percentages of CD3+ cells within total lymphocytes. Showing absolute counts, percentages and subsets of CD4+ and CD8+ T cells. T cell subsets were defined as; CD45RA+ CD27+ naive, CD45RA− CD27+ memory, and CD45RA+/− CD27− effector cell populations.

*** As a % of CD3+CD4+ T cells ** As a percentage of CD3+CD8+ T cells**

|  | **Patient #1** | **Patient #2** | **Patient #3** | **Patient #4** | **Patient #5** |
| --- | --- | --- | --- | --- | --- |
| **CD3+ abs (x10^9^/L)** | 1150 | 1551 | 2790 | 1829 | 1800 |
| **CD3+ (% of lymfocytes)** | 73,5 | 80,1 | 83,4 | 87,7 | 81.2 |
| **CD4+ abs (x10^9^/L)** | 527 | 1257 | 754 | 1661 | 1050 |
| **CD4+ (% of CD3+)** | 33,7 | 64,9 | 22,5 | 77,3 | 50.4 |
| **CD4+RA+CD27+ *** | 39 | 40,7 | 19 | 8 | 26.3 |
| **CD4+RA-CD27+ *** | 39,7 | 40 | 60,7 | 80,1 | 55.1 |
| **CD4+RA-CD27- *** | 20,4 | 4,1 | 18,1 | 11 | 13.1 |
| **CD4+RA+CD27- *** | 0,9 | 15,2 | 2,2 | 0,9 | 5.5 |
| **CD8+ abs (x10^9^/L)** | 542 | 284 | 2006 | 208 | 750 |
| **CD8+ (% of CD3+)** | 34,7 | 14,7 | 60 | 10 | 30.6 |
| **CD8+RA+CD27+ **** | 16,4 | 36,4 | 7,8 | 22 | 20.2 |
| **CD8+RA-CD27+ **** | 27,7 | 25,5 | 45,7 | 64.3 | 40.3 |
| **CD8+CD27- **** | 55,9 | 38,1 | 46,5 | 13,7 | 39.5 |
